# Dietary acrylamide and incident osteoporotic fractures: an 8-year prospective cohort study

**DOI:** 10.1007/s40520-022-02214-9

**Published:** 2022-08-13

**Authors:** Nicola Veronese, Francesco Bolzetta, Chiara Cacco, Alberto Cester, Lee Smith, Jacopo Demurtas, Cyrus Cooper, Renè Rizzoli, Maria Gabriella Caruso, Maria Notarnicola, Jean-Yves Reginster, Stefania Maggi, Mario Barbagallo, Mike Trott, Ligia J. Dominguez

**Affiliations:** 1 Geriatric Unit, Department of Internal Medicine and Geriatrics, University of Palermo, via del Vespro, 141, 90127 Palermo, Italy; 2 Medical Department, Geriatric Unit, Azienda ULSS (Unità Locale Socio Sanitaria, Dolo-Mirano District, 3 “Serenissima”, Dolo, Italy; 3 University of Padova, Oncology, Padua, Italy; 4 The Cambridge Centre for Sport and Exercise Sciences, Anglia Ruskin University, Cambridge, CB1 1PT UK; 5 Primary Care Department, Azienda USL Toscana Sud Est, Grosseto, Italy; 6 MRC Lifecourse Epidemiology Unit, University of Southampton, Southampton General Hospital, Southampton, UK; 7 NIHR Musculoskeletal Biomedical Research Unit, University of Oxford, Oxford, UK; 8 Division of Bone Diseases, Geneva University Hospitals and Faculty of Medicine, Geneva, Switzerland; 9 National Institute of Gastroenterology, Research Hospital, IRCCS De Bellis, Castellana Grotte, Bari, Italy; 10 WHO Collaborating Centre for Public Health Aspects of Musculoskeletal Health and Aging, Liège, Belgium; 11 Department of Public Health, Epidemiology and Health Economics, University of Liège, CHU Sart Tilman B23, Liège, Belgium; 12 Aging Branch, Neuroscience Institute, National Research Council, 35121 Padua, Italy; 13 Vision and Eye Research Institute, Faculty of Health and Medical Sciences, Anglia Ruskin University, Cambridge, UK; 14 Faculty of Medicine and Surgery, University of Enna “Kore”, Enna, Italy

**Keywords:** Acrylamide, Fracture, Osteoporosis, Osteoarthritis initiative

## Abstract

**Background:**

Acrylamide, a component of fried foods, has been associated with several negative health outcomes. However, the relationship between dietary acrylamide and osteoporotic fractures has been explored by a few cross-sectional studies.

**Aims:**

To investigate if dietary acrylamide is associated with the onset of fractures in North American participants at high risk/having knee osteoarthritis (OA), over 8 years of follow-up.

**Methods:**

A Cox’s regression analysis, adjusted for baseline confounders was run and the data were reported as hazard ratios (HRs) and 95% confidence intervals (CIs). Dietary acrylamide intake was assessed at the baseline using a food frequency questionnaire and categorized in tertiles (T), whilst fractures’ history was recorded using self-reported information.

**Results:**

Altogether, 4,436 participants were included. Compared to participants with lower acrylamide intake (T1; < 3,313 μg), those with a higher acrylamide intake (T3; > 10,180 μg) reported a significantly higher risk of any fracture (HR = 1.37; 95% CI 1.12–1.68; *p* for trend = 0.009), forearm (HR = 1.73; 95% CI 1.09–2.77; *p* for trend = 0.04), spine (HR = 2.21; 95% CI 1.14–4.31; *p* for trend = 0.04), and hip fracture (HR = 4.09; 95% CI 1.29–12.96; *p* for trend = 0.046).

**Conclusions:**

Our study is the first to report that high dietary acrylamide may be associated with an increased risk of osteoporotic fractures.

## Introduction

Acrylamide is a vinyl monomer principally derived from chemical industries to produce polymers for water treatment, oil drilling, paper making and mineral processing [1]. Acrylamide was firstly evaluated by the International Agency for Research on Cancer and identified as potentially carcinogenic to humans [2]. As a food component, acrylamide can be formed during the thermal processing of carbohydrate-rich foods, especially deep-frying, oven-baking, and roasting [3].

The amount of acrylamide in cooked foods is determined by cooking temperature and time and the quantity of reducing sugar and asparagine in raw foods [4]. The World Health Organization (WHO) attempted to evaluate the average exposure to acrylamide in foods, however, this is difficult to measure with traditional instruments available in nutritional research [5]. At the same time, increasing research is reporting that higher dietary acrylamide intake could be associated with a higher risk of cancer, in particular genito-urinary [6, 7] or negative outcomes in pregnancy and in newborns [8, 9].

Recently Pan et al. found that acrylamide exposure in animal cell lines led to oxidative stress as shown by a significant increase in reactive oxygen species (ROS) and malondialdehyde (MDA) levels and glutathione (GSH) reduction, all factors associated with osteoporosis and fractures in older people [10]. Inflammatory response was observed based on dose-dependent levels of dietary acrylamide in relation to pro-inflammatory cytokines tumor necrosis factor-α (TNF-α) and interleukin 6 (IL-6) [10]. In addition, ACR-activated nuclear transcription factor E2-related factor 2 (Nrf2) and nuclear factor-κB (NF-κB) signaling pathways were also observed [6]. Moreover, analogous effects on inflammation parameters were reported in recent literature [11].

There is increasing attention to the role of oxidative stress in the pathogenesis of osteoporosis, in the context of several others classically known risk factors such as early menopause, low weight, the use of corticosteroids, smoking [12]. Cervellati et al. for example, suggested that estrogen withdrawal, typical of menopause, might contribute to increased bone vulnerability, with an increased risk of osteoporosis development [13]. Moreover, recent literature proposed a shift from the “estrogen-centric” account of the pathogenesis of osteoporosis, to another model where oxidative stress was also involved. In 2016 a meta-analysis including 17 observational studies [14] suggested a significant association between oxidative stress and osteoporosis, with the disequilibrium of ROS and antioxidant system as contributors to functional and structural remodeling of the bone. In animal models, some recent studies reported different effects on compact and trabecular bone tissue to a single oral acrylamide dose administration, with loss of thickness in cortical bone, through an increase in oxidative stress and inflammation [15, 16]. However, data regarding the association between dietary acrylamide and fractures in human beings are still missing, whilst increasing literature has reported that to follow a healthy diet pattern was associated with a lower incidence of fractures [17]. Finally, osteoporosis is often associated with important sequelae (such as disability and sarcopenia) [18] that further underlines the need of stratifying patients according to relevant risk factors [19, 20].

Having this in mind, the aim of the present study was to investigate the relationship between baseline dietary acrylamide intake and fractures development, over 8 years of follow-up. It is hypothesized that people with a higher intake of acrylamide would be at higher risk of osteoporotic fractures.

## Materials and methods

### Data source and participants

Information from the Osteoarthritis Initiative (OAI) database (https://www.niams.nih.gov/grants-funding/funded-research/osteoarthritis-initiative) were used for the present analyses. Potential participants were included from 4 different sites in the United States of America (Baltimore, MD; Pittsburgh, PA; Pawtucket, RI; and Columbus, OH) between February 2004 and May 2006.

All participants provided written informed consent. The OAI study was given full ethical approval by the institutional review board of the OAI Coordinating Center, at the University of California in San Francisco. Note that because this analysis was based on publicly available data, local ethical approval was not required.

### Inclusion and exclusion criteria

The participants of the OAI must meet one of the following criteria: (i) being overweight; (ii) had a previous knee injury or surgery; (iii) had knee pain during the past year; (iv) or had a parent or sibling who had a knee replacement. Conversely, they were excluded if they suffer on rheumatoid arthritis, joint replacements in both knees, unable to walk without assistance or unable to undergo magnetic resonance of the knees.

### Exposure

Dietary acrylamide intake was obtained through a food frequency questionnaire (FFQ) recorded during the baseline visit of the OAI. Dietary pattern was analyzed with the use of a validated tool called the Block Brief 2000 food-frequency questionnaire. This food-frequency questionnaire contains a list of 70 items and is designed to assess usual food and beverage consumption over the past year. Frequency of consumption of the 70 foods was reported at 9 levels of intake from never to every day. In addition, there were 7 dietary behavior queries on food preparation methods and fat intake, 1 on fiber intake, and 13 on vitamin and mineral intakes [21]. The product yearly frequency by portions’ size, contained in the FFQ, was categorized into standard portions (median value) [22] and then grams. The presence of acrylamide in foods was estimated using 2015 data from the Food and Drugs Administration (FDA) website (https://www.fda.gov/food/chemicals/survey-data-acrylamide-food). The general limit of quantitation (LOQ) of the method used by the FDA is 10 ppb. The data for this work regarding dietary acrylamide were reported as μg consumed in one year by each subject. The participants were divided in tertiles according to their dietary acrylamide intake, i.e., < 3313, between 3314 and 10,180, and > 10,180 μg, respectively.

### Outcomes

The presence of fractures at baseline and during follow-up was ascertained through the self-reported history of fractures at the most common sites, i.e., hip, spine and forearm. The primary outcome was considered the incidence of any fracture, the osteoporotic specific sites (hip, forearm, spine) were considered as secondary outcomes. The presence of fractures in the OAI was recorded, other than the first evaluation, after 1, 2, 3, 4, 6 and 8 years from baseline.

### Covariates

In these analyses, several covariates were identified as potential confounding factors, based on the literature available regarding dietary patterns and osteoporosis fractures. These included: age; gender; calorie intake (in Kcal); body mass index (BMI); depressive symptoms assessed with the Center for Epidemiologic Studies Depression Scale (CES-D) [23]; physical activity evaluated using the total score for the Physical Activity Scale for the Elderly scale (PASE) [24]; race; smoking habit; educational attainment level (college or higher vs. others); yearly income (< or ≥ $50,000 or missing data); the modified Charlson Comorbidity Index score [25]; use of bisphosphonates/parathyroid hormones analogues (anti-osteoporotic medications) or estrogens/testosterone for menopausal symptoms; the presence of osteoporotic fractures at baseline; and dietary fibers in fruits and vegetables; dietary intake of vitamin D, calcium, proteins; number of alcoholic drinks during a typical week.

### Statistical analyses

Continuous variables were normally distributed according to the Kolmogorov–Smirnov test. Therefore, data were shown as means and standard deviation values (SD) for quantitative measures. Percentages were used for discrete variables. Levene’s test was used to test the homoscedasticity of variances and, if its assumption was violated, Welch’s ANOVA was used. P-values were calculated using the Jonckheere-Terpstra test [26] for continuous variables and the Mantel–Haenszel Chi-square test for categorical variables.

For assessing the relationship between dietary acrylamide intake and the onset of fractures during follow-up, a Cox’s regression analysis was done. The incidence of fractures during the follow-up, evaluated at visit number 1, 3, 5, 6, and 8 was graphically reported using survival curves. Deceased people were censored. Dietary acrylamide was categorized into tertiles, with Tertile 1 (the lowest intake) representing the reference group. Multi-collinearity among covariates, cited before, was assessed using variance inflation factor (VIF) [27], taking a cut-off of 2 as the criterion for exclusion, even if no covariates met this criterion. Adjusted hazard ratios (HR) and 95% confidence intervals (CI) were reported to estimate the presence and the strength of the associations between dietary acrylamide intake and incident osteoporotic fractures. P values for trend were calculated across dietary acrylamide tertiles using the Wald test, based on a score derived from the median value of each baseline tertile [28].

Some sensitivity analyses were conducted for showing the interaction between dietary acrylamide intake and some selected variables (i.e., age below or more/equal than 65 years, overweight/obese (≥ 25 kg/m^2^) vs. normal weight (18.5 kg/m^2^ < BMI < 25 kg/m^2^), yearly income, gender, race, education, smoking habits, presence of fractures at the baseline, use of anti-osteoporotic medications) in the association with incident fractures, only presence of fractures at the baseline and use of anti-osteoporotic medications emerged as effect modifiers of the associations examined (*p* < 0.0001 for the interaction).

All analyses were performed using the SPSS 20.0 for Windows (SPSS Inc., Chicago, Illinois). All statistical tests were two-tailed and statistical significance was assumed for a *p*-value < 0.05.

## Results

### Study participants

In 4796 initially included individuals, 243 reported < than 500 or > 5000 kcal (not reliable calorie intake) or did not have any information regarding FFQ and 117 were lost at follow-up (i.e. did not have information regarding fractures during the follow-up period), leaving 4436 participants included for this research. The 360 people not included were significantly older and more frequently females than people included.

### Baseline analyses

Altogether, 4436 participants (of them 2578 women) with a mean age of 61.3 ± 9.1 (range: 45–79) years were included in the analysis. The mean acrylamide intake, by year, was 13,985 ± 23,863 (range: 0–364,438) μg (mean 38.3 μg/day).

The baseline characteristics, by dietary acrylamide intake divided into tertiles, are shown in Table 1. People having a greater dietary acrylamide intake (T3) were significantly younger, more frequently males, less educated, had a significant higher calorie intake and were more frequently obese and sedentary than those introducing less acrylamide with their diet (T1) (Table 1). People introducing more acrylamide with their diet reported a significantly higher consumption of proteins and fibers, derived from vegetables, as well as calcium. People with the highest tertiles of dietary acrylamide intake used less frequently anti-osteoporotic medications (7.0 in T3 vs. 19.0% in T1, *p* < 0.0001) and they reported more frequently osteoporotic fractures (19.8 in T3 vs. 16.3% in T1, *p* = 0.01) (Table 1).

**Table 1:** Descriptive baseline characteristics by dietary acrylamide intake

Parameter	T1^†^ (*n* = 1488)	T2^†^ (*n* = 1485)	T3^†^ (*n* = 1463)	*p*-value^*^
Median dietary acrylamide intake (μg)	1616	6004	19,935	–
Mean (SD) dietary acrylamide intake (μg)	1473 (882)	6617 (2469)	34,192 (33,127)	–
Mean (SD) μg/kg bodyweight per day	20 (12)	84 (36)	398 (374)	–
Range (μg)	0–3313	3314–10,180	10,181–364,438	–
Age (SD)	63.5 (9.1)	61.4 (9.1)	59.0 (8.8)	< 0.0001
Female gender (%)	71.1	59.6	43.4	< 0.0001
Calorie intake (Kcal) (mean, SD)	1200 (446)	1338 (492)	1712 (628)	< 0.0001
Vitamin D (UI) (mean, SD)	146 (112)	137 (103)	142 (108)	0.06
Calcium (mg) (mean, SD)	661 (343)	657 (331)	696 (335)	0.003
Proteins (g) (mean, SD)	53 (22)	57 (23)	71 (28)	< 0.0001
Vegetable fibers (g)	7.8 (4.9)	7.1 (4.1)	8.0 (4.8)	< 0.0001
Number of alcoholic drinks in a week (mean, SD)	1.7 (1.4)	1.7 (1.5)	1.8 (1.5)	0.14
BMI (Kg/m^2^) (mean, SD)	27.4 (4.6)	28.7 (4.7)	29.8 (4.8)	< 0.0001
CESD (points) (mean, SD)	6.4 (69)	6.6 (6.9)	6.8 (6.9)	0.24
PASE (points) (mean, SD)	169 (85)	160 (81)	154 (79)	< 0.0001
Charlson comoribidity index (points) (mean, SD)	0.40 (0.89)	0.38 (0.81)	0.39 (0.83)	0.77
Yearly income (> 50,000 $)	39.0	35.8	37.3	0.96
Whites (%)	84.7	82.1	74.1	< 0.0001
College or higher (%)	33.3	30.2	27.6	< 0.0001
Current/previous smokers (%)	53.6	53.7	50.2	0.07
Use of anti-osteoporotic medications (%)	19.0	11.1	7.0	< 0.0001
Osteoporotic fractures at baseline (%)	16.3	17.2	19.8	0.01

*SD* standard deviation, *BMI* body mass index, *CESD* center for epidemiologic studies depression scale, *PASE* physical activity scale for elderly

*p-values for trend were calculated using the Jonckheere-Terpstra test for continuous variables and the Mantel–Haenszel Chi-square test for categorical variables

†The cut-offs used for creating the tertiles were 3313 and 10,180 μg, respectively

### Follow-up analyses and incident fractures onset

During eight years of follow-up, 789 osteoporotic fractures were observed. As shown in Table 2, people with higher dietary acrylamide intake reported a significant higher incidence of fractures compared to those with lower dietary acrylamide intake (34, 95% CI 30–38 in T3 vs. 25, 95% CI 22–29 in T1).

**Table 2:** Association between baseline acrylamide intake and incident fracture

Acrylamide intake^†^	Cases	Participants	Incidence rate (95%CI) per 1000-person years	Person-years	Age- adjusted HR (95%CI) *p*-value	Fully-adjusted HR^*^ (95%CI) *p*-value
Any fracture						
T1	295	1488	25 (22–29)	9074	1 [ref] *P* for trend = 0.04	1 [ref] *P* for trend = 0.009
T2	269	1485	30 (26–34)	9175	1.14 (0.95–1.36) *p* = 0.16	1.24 (1.02–1.49) *p* = 0.03
T3	225	1463	34 (30–38)	9416	1.26 (1.05–1.50) *p* = 0.01	1.37 (1.12–.68) *p* = 0.002
Forearm fracture						
T1	52	1488	4 (2–5)	2822	1 [ref] *P* for trend = 0.12	1 [ref] *P* for trend = 0.04
T2	55	1485	6 (4–8)	3143	1.51 (0.97–2.36) *p* = 0.07	1.82 (1.10–3.00) *p* = 0.02
T3	33	1463	6 (5–8)	4232	1.53 (0.99–2.37) *p* = 0.06	1.73 (1.09–2.77) *p* = 0.02
Spine fracture						
T1	36	1488	2 (1–3)	1231	1 [ref] *P* for trend = 0.12	1 [ref] *P* for trend = 0.04
T2	22	1465	2 (1–4)	1343	1.14 (0.60–2.15) *p* = 0.69	1.40 (0.72–2.74) *p* = 0.32
T3	17	1463	4 (3–6)	2841	1.74 (0.97–3.14) *p* = 0.07	2.21 (1.14–4.31) *p* = 0.02
Hip fracture						
T1	22	1488	1 (0.1–1.5)	1023	1 [ref] *P* for trend = 0.03	1 [ref] *P* for trend = 0.046
T2	18	1485	2 (1–3)	2045	3.67 (1.23–10.95) *p* = 0.02	3.84 (1.25–11.82) *p* = 0.02
T3	4	1463	3 (2–4)		4.33 (1.48–12.73) *p* = 0.008	4.09 (1.29–12.96) *p* = 0.02

^*^Data are reported as hazard ratios (HRs) with their 95% confidence intervals (CIs), adjusted for: age, gender, calorie intake, body mass index, Center for Epidemiologic Studies Depression Scale, Physical Activity Scale for Elderly, Charlson comoribidity index, yearly income, race, educational level, smoking status, use of anti-osteoporotic medications, presence of osteoporotic fractures at baseline, dietary fibers in fruits and vegetables; dietary intake of vitamin D, calcium, proteins; number of alcoholic drinks during a typical week. ^†^The cut-offs used for creating the tertiles were 3313 and 10,180 μg, respectively

In multivariable analyses, after taking into account 18 different potential confounders at baseline, compared to participants with lower acrylamide intake (T1), those with a higher acrylamide intake (T3) reported a significant higher risk of any fracture (HR = 1.37; 95% CI 1.12–1.68; *p* = 0.002) (*p* for trend = 0.009). Similar results were observed taking, as outcomes, specific-site fractures, including forearm fracture (HR = 1.73; 95% CI 1.09–2.77) (*p* for trend = 0.04), spine fracture (HR = 2.21; 95% CI 1.14–4.31; *p* for trend = 0.04), and hip fracture (HR = 4.09; 95% CI 1.29–12.96; *p* for trend = 0.046) (Table 2).

In sensitivity analyses, it was found that the association between dietary acrylamide intake and incident fractures was stronger in people with the presence of an osteoporotic fracture at the baseline (T3: HR = 1.84; 95% CI 1.18–2.22, *p* = 0.002) (*p* for interaction < 0.0001) and in those not taking any anti-osteoporotic medication (T3: HR = 1.36; 95% CI 1.14–1.84; *p* = 0.001) (*p* for interaction < 0.0001). The *p* for the interaction sex by baseline dietary acrylamide intake was 0.48, indicating no difference between males and females in reporting fractures related to this factor.

## Discussion

In the present study, involving a large cohort of adults with or at high risk of knee OA residing in North America, it was found that people with higher dietary acrylamide intake have an increased risk of osteoporotic fractures, over eight years of follow-up. This finding remained unaltered after adjustment for several potential confounders and when considering site-specific fractures. Finally, effects were stronger in people having already had a diagnosis of osteoporosis at baseline and in those not taking any medication for this condition.

In a review including 101 studies and investigating the dietary intake of acrylamide, the authors found mean/median values of acrylamide in North American people lower than those found in the OAI, probably because in the review general populations are reported whilst in the OAI obese/overweight people are included [2]. At baseline people having a higher dietary acrylamide intake were significantly younger, more frequently males, less educated, had a significantly higher calorie intake and were more frequently obese and sedentary than those introducing less acrylamide with their diet. These findings were expected, since in this study a high acrylamide intake reflects a propensity to an unhealthy diet, rich in fried and roasted food, common in North American obese people and in younger populations [29–31]. Moreover, it has been reported that North American women more frequently consume healthy food such as vegetables and fruits than men [32] and that there is a strong consistent relationship between low socio-economic factors, as low scholar education, in early life and increased obesity in adulthood [33]. Furthermore, diets rich in fruit and vegetables have been shown to decrease the risk of the incidence of hip fractures [34, 35].

To the best of our knowledge, this is the only study evaluating the relationship between the dietary intake of acrylamide and osteoporotic fractures in human beings. There are only two studies in animals that evaluated the effects of an acute oral administration of acrylamide on bone microstructure analysis, finding a significant loss of thickness in femoral cortical bone of mice after oral acrylamide exposure [15, 16]. More recently, another in the animal study reported that the exposure to acrylamide resulted in skeletal structure malformation in zebrafish and rat embryos and that the toxicity of acrylamide might translate to the next generation [36]. In particular, the term cortical osteoporosis includes mechanically inappropriate cortical thinning as well as excessive porosity and is almost certainly a relevant factor in hip fracture in older people [37, 38]. In normal conditions, cortical bone tissue deforms under a mechanical load and then regains its previous anatomical form, the elasticity of cortical bone tissue is a measure of resistance to deformation [39]. Another important point that future research should verify is that the association between dietary acrylamide and incident fractures is stronger in people already having osteoporosis. With the data available so far, one can only give a tentative suggestion in limiting the introduction of fried foods in those with osteoporosis as this may result in a higher vulnerability to fractures.

From a pathophysiological point of view, it is possible that ROS could be the link between dietary acrylamide intake and osteoporosis. As already pointed out, acrylamide exposure has been shown to lead to oxidative stress characterized by a significant increase in reactive oxygen species [10]. In a meta-analysis on the association between oxidative stress and osteoporosis, increased levels of homocysteine and nitric oxide in osteoporotic participants and decreased levels of folates and total antioxidant power, along with the lower activity of superoxide dismutase and glutathione peroxidase, were reported suggesting a significant association between oxidative stress and osteoporosis [14].

The present findings have potentially important clinical implications, not only limited to acrylamide intake and osteoporotic fractures. Major food sources of acrylamide are deep fried and roasted food, commonly consumed as a part of an unhealthy diet. Conversely, a diet with high consumption of vegetables has been related to have a protective effect against osteoporosis and fractures [40]. However, it should be noted that vegetarians (in particular vegans) are exposed to a higher risk of fractures than omnivores, possibly due to a lack of other important nutrients, including calcium and protein [41, 42].

Finally, it is important to discuss how to limit dietary acrylamide intake. The first, obvious, intervention is to limit those foods rich in acrylamide such as fried fries and the other foods undergoing the Maillard reaction [43]. Another method to limit the production of dietary acrylamide is adding some nutrients, such as vitamins, minerals, amino acids, and in cooking oils, even if these findings are still exploratory [43].

Findings from this study should be interpreted in light of its limitations. First, dietary acrylamide was assessed only at baseline; therefore, it was not possible to assess whether changes in acrylamide consumption over time can modify the results. In this regard, we also note that its estimation can be difficult using the FFQ or other traditional nutritional tools. In particular, even if FFQ is the most used tool in nutritional epidemiology, the assessment of dietary acrylamide using this tool is not validated, and therefore our results should be treated with caution. Second, the information regarding fractures was self-reported in the OAI and we do not have specific information regarding if a fracture was due to osteoporosis or other causes. In this regard, some studies showed that for clinical fractures the accuracy of self-reported fractures is accurate and similar to radiological records, but probably there is an underestimation of some non-clinical fractures, especially vertebral ones [44, 45]. Third, no data about bone mineral density, renal function and vitamin D levels are available and this could introduce another bias in the findings. Fourth, the OAI selectively oversamples individuals who are obese. Therefore, it is likely that the simple adjustment for BMI might be unable to remove residual confounding attributable to adiposity in this specific population. Fifth, the small number of cases in the site-specific fracture, such as 4 cases in T3 for hip fracture, indicate to take cautiously the findings regarding specific-sites fractures. Furthermore, the OAI included only participants with knee osteoarthritis or at high risk of this condition, likely introducing a selection bias, and limiting the generalizability of results. Finally, no information on the menopausal status of the women in the study was available, therefore the effect of menopausal status (and associated hormone replacement therapies) are unknown.

In conclusion, higher dietary acrylamide intake was significantly associated with a higher risk of osteoporotic fractures, also after accounting for potential confounders, suggesting a role for this food contaminant as a possible risk factor for osteoporosis. Overall, people with introducing more acrylamide with their diet reported a significantly higher risk of any site and specific site (forearm, hip, vertebral) fractures. Our study further underlines the importance of consuming low amounts of acrylamide with a diet that seems associated with several negative outcomes in human beings including cancer and now osteoporotic fractures.
